# Kinematic characteristics during gait in frail older women identified by principal component analysis

**DOI:** 10.1038/s41598-022-04801-2

**Published:** 2022-01-31

**Authors:** Wakako Tsuchida, Yoshiyuki Kobayashi, Koh Inoue, Masanori Horie, Kumiko Yoshihara, Toshihiko Ooie

**Affiliations:** 1 Department of Life Science and Biotechnology, Health and Medical Research Institute, National Institute of Advanced Industrial Science and Technology (AIST), 2217-14 Hayashi-cho, Takamatsu, Kagawa 761-0395 Japan; 2 Human Augmentation Research Center, National Institute of Advanced Industrial Science and Technology (AIST), Kashiwa II Campus, University of Tokyo, 6-2-3 Kashiwanoha, Kashiwa, Chiba 277-0882 Japan; 3 Faculty of Engineering and Design, Kagawa University, Hayashi-cho 2217-20, Takamatsu, Kagawa 761-0396 Japan

**Keywords:** Geriatrics, Biomarkers, Health care

## Abstract

Frailty is associated with gait variability in several quantitative parameters, including high stride time variability. However, the associations between joint kinematics during walking and increased gait variability with frailty remain unclear. In the current study, principal component analysis was used to identify the key joint kinematics characteristics of gait related to frailty. We analyzed whole kinematic waveforms during the entire gait cycle obtained from the pelvis and lower limb joint angle in 30 older women (frail/prefrail: 15 participants; non-frail: 15 participants). Principal component analysis was conducted using a 60 × 1224 input matrix constructed from participants’ time-normalized pelvic and lower-limb-joint angles along three axes (each leg of 30 participants, 51 time points, four angles, three axes, and two variables). Statistical analyses revealed that only principal component vectors 6 and 9 were related to frailty. Recombining the joint kinematics corresponding to these principal component vectors revealed that frail older women tended to exhibit greater variability of knee- and ankle-joint angles in the sagittal plane while walking compared with non-frail older women. We concluded that greater variability of knee- and ankle-joint angles in the sagittal plane are joint kinematic characteristics of gait related to frailty.

## Introduction

Frailty is associated with an increased risk of adverse consequences, including functional decline, deterioration, multiple hospitalizations and death, and is an important public health concern in aging societies^1–6^. Clinical literature over the past several decades has identified clear sex differences in frailty, typically reporting that women have a higher prevalence and risk of frailty^7–11^. Women are also reported to have a higher prevalence of muscle weakness, lower bone density and a higher risk of future fractures associated with a decrease in female hormone levels with menopause, and are at a higher risk of decline in walking ability and becoming bedridden from frailty than males^13,70–73^. To prevent frailty, it is important to detect symptoms of frailty before the condition becomes severe, so that appropriate interventions can be carried out^12^. Preclinical changes in gait have been reported as an early sign of frailty, for both males and females^13–24^. Therefore, comparison of gait characteristics in healthy and frail older women is necessary for gaining a detailed understanding of the frailty-related decrease in walking ability among older women.

Gait variability is associated with frailty status^17^. Previous studies have consistently reported that, compared with healthy older adults, frail older adults tend to exhibit greater within-participant variability on various gait related parameters, including stride length, step width and cadence, using pressure sensitive walkways or accelerometry^17,18,25,26^. However, the way in which the joint kinematics of the lower extremities during walking are associated with increased gait variability in frailty has not yet been thoroughly investigated. In addition, to the best of our knowledge, no previous study has evaluated joint angles or the variability of the lower extremities during the entire gait cycle in relation to frailty status. Detailed investigation of the relationships between frailty status and gait characteristics, including variability in joint kinematics while walking, could be helpful for understanding the mechanisms of increased gait variability and decreased walking ability in frail older adults. Moreover, elucidating this issue could inform the development of better indicators for early detection of frailty and early preventive measures.

In the last several decades, the effects of frailty on various gait variables, such as walking speed, stride length, step width and cadence have been investigated^17–31^. However, these previous studies of gait in frail older people have focused on variables and discrete time points selected by researchers. An advantage of traditional biomechanical analysis methods (e.g., analysis of several discrete variables, such as peak angles, together with statistical hypothesis tests, such as t-tests or analysis of variance) is that they allow for detailed comparisons of selected variables between different groups, and investigation of relevant factors. However, the disadvantage of these methods is that they may not be able to detect crucial information in large portions of unanalyzed data^32^. In this regard, principal component analysis has recently attracted increasing interest in biomechanical studies because of its usefulness in identifying the movement characteristics of various groups under a range of conditions using waveforms of the entire time series data set in a comprehensive manner^32–42^. Principal component analysis is a multivariate statistical technique that summarizes the information conveyed by a large number of correlated variables using a smaller number of uncorrelated variables (principal components). Principal component analysis generates principal component vectors and a set of principal component scores for each principal component vector. Each principal component vector corresponds to an axis of variance, and a principal component score is a projection of the input data onto each principal component vector. The waveforms related to each principal component vector can be reconstructed by adding and subtracting the principal component scores. Therefore, the use of this principal component analysis-based approach could enable increased understanding of walking characteristics and joint kinematics among frail older women throughout the entire gait cycle.

The purpose of the current study was to compare the key characteristic features of joint kinematics during the entire gait cycle in frail older women and non-frail older women using principal component analysis. To understand the gait characteristics of frail older women, we also compared basic spatiotemporal parameters of the gait cycle: walking speed, stride length, stride time, and stance time percentage. Previous studies of combined samples of women and males have reported that frail older adults exhibit increased variability in stride time and length compared with non-frail older adults^17,18,25^. Therefore, we hypothesized that walking in frail older women involves increased joint angle variability of the lower extremities during the swing phase of the gait cycle compared with that in non-frail older women.

## Results

Participants’ demographic data (means and standard deviations [SDs]) are presented in Table 1. We defined frailty based on criteria developed by Fried et al.^6^. Thirteen participants were classified as prefrail, two were classified as frail and 15 were classified as non-frail. There were no significant differences in basic information between frail/prefrail participants and non-frail participants.

**Table 1:** Demographics of participants.

Variables	Non-frail (n = 15)	Frail/prefrail (n = 15)	*p*-value
	(Mean ± SD)	(Mean ± SD)	
Age (years)	69.3 ± 5.2	69.4 ± 4.6	0.94
Height (cm)	153.8 ± 4.4	151.9 ± 3.8	0.23
Body mass (kg)	53.4 ± 6.0	53.9 ± 5.5	0.83
Grip (kg)	22.7 ± 3.1	21.2 ± 4.8	0.32
SMI	6.03 ± 0.5	6.06 ± 0.6	0.87
**Frailty criteria**			
Weight loss (n)	0	4	
Slowness (n)	0	1	
Weakness (n)	0	5	
Exhaustion (n)	0	0	
Low activity (n)	0	12	
**Sarcopenia criteria**			
Sarcopenia (n)	0	2	
Severe sarcopenia (n)	0	1	

*SMI* skeletal muscle mass index.

Principal component analysis revealed that 26 extracted principal component vectors explained more than 84% of the joint movement patterns. The explained variance, means, and SDs of the principal component scores by group are shown in Table 2. Univariate analysis (independent t-tests) revealed significant differences between frail/prefrail participants and non-frail participants on principal component vectors 6 (*p* = 0.024, *d* = 0.60) and 9 (*p* = 0.001, *d* = 0.72) and explained 4.12% and 3.33% of the total variance, respectively.

**Table 2:** Results of main principal component analysis.

	PCV1	PCV2	PCV3	PCV4	PCV5	PCV6
Explained variance (%)	11.3	9.24	7.97	6.52	4.85	4.12
Cumulative (%)	11.3	20.5	28.5	35.0	39.8	43.9
Non-frail (mean ± SD)	0.04 ± 1.13	0.00 ± 0.78	− 0.06 ± 0.95	0.27 ± 1.03	0.12 ± 0.97	− 0.29 ± 0.99
Frail/prefrail (mean ± SD)	− 0.04 ± 0.87	0.00 ± 1.19	0.06 ± 1.06	− 0.15 ± 0.93	− 0.12 ± 1.03	0.29 ± 0.93
*p*-value	0.77	1.00	0.64	0.11	0.36	0.02*
Cohen’s *d*	0.07	0.00	0.12	0.42	0.24	0.60

	PCV7	PCV8	PCV9	PCV10	PCV11	PCV12
Explained variance (%)	3.90	3.45	3.33	3.03	2.64	2.45
Cumulative (%)	47.8	51.3	54.6	57.7	60.3	62.7
Non-frail (mean ± SD)	0.09 ± 1.01	− 0.23 ± 0.90	− 0.34 ± 0.91	0.07 ± 1.08	0.06 ± 0.76	0.09 ± 1.32
Frail/prefrail (mean ± SD)	− 0.09 ± 1.00	0.23 ± 1.06	0.34 ± 0.99	− 0.07 ± 0.92	− 0.06 ± 1.21	− 0.09 ± 0.52
*p*-value	0.51	0.07	0.00*	0.57	0.66	0.50
Cohen’s *d*	0.17	0.47	0.71	0.15	0.12	0.18

	PCV13	PCV14	PCV15	PCV16	PCV17	PCV18
Explained variance (%)	2.39	2.11	1.89	1.72	1.59	1.54
Cumulative (%)	65.1	67.2	69.1	70.8	72.4	74.0
Non-frail (mean ± SD)	− 0.01 ± 0.99	− 0.02 ± 1.14	− 0.16 ± 0.98	0.11 ± 1.02	− 0.11 ± 0.96	− 0.17 ± 1.09
Frail/prefrail (mean ± SD)	0.01 ± 1.03	0.02 ± 0.86	0.16 ± 1.01	− 0.11 ± 0.99	0.11 ± 1.04	0.27 ± 0.87
*p*-value	0.91	0.90	0.22	0.42	0.40	0.09
Cohen’s *d*	0.03	0.03	0.32	0.21	0.22	0.45

	PCV19	PCV20	PCV21	PCV22	PCV23	PCV24
Explained variance (%)	1.48	1.43	1.41	1.24	1.20	1.18
Cumulative (%)	75.5	76.9	78.3	79.5	80.7	81.9
Non-frail (mean ± SD)	0.16 ± 1.08	0.24 ± 1.07	0.16 ± 0.92	0.00 ± 1.11	0.05 ± 1.02	0.12 ± 0.98
Frail/prefrail (mean ± SD)	− 0.16 ± 0.90	− 0.24 ± 0.87	− 0.16 ± 1.06	0.00 ± 0.90	− 0.05 ± 0.99	− 0.12 ± 1.02
*p*-value	0.22	0.06	0.21	1.00	0.71	0.38
Cohen’s *d*	0.32	0.50	0.33	0.00	0.10	0.23

	PCV25	PCV26				
Explained variance (%)	1.08	1.04				
Cumulative (%)	83.0	84.0				
Non-frail (mean ± SD)	0.12 ± 0.93	− 0.02 ± 1.05				
Frail/prefrail (mean ± SD)	− 0.12 ± 1.07	0.02 ± 0.96				
*p*-value	0.36	0.89				
Cohen’s *d*	0.24	0.04				

Principal component analysis was applied to the correlation matrix of 1224 variables (i.e., intra-participant mean and standard deviation for 51 time points, four angles in three axes) calculated from the 60 data sets (each leg of 30 participants).

*PCV* principal component vector.

The “*” symbol indicates significant differences between the non-frail and frail/prefrail groups (*p < 0.05).

**Figure 1:**
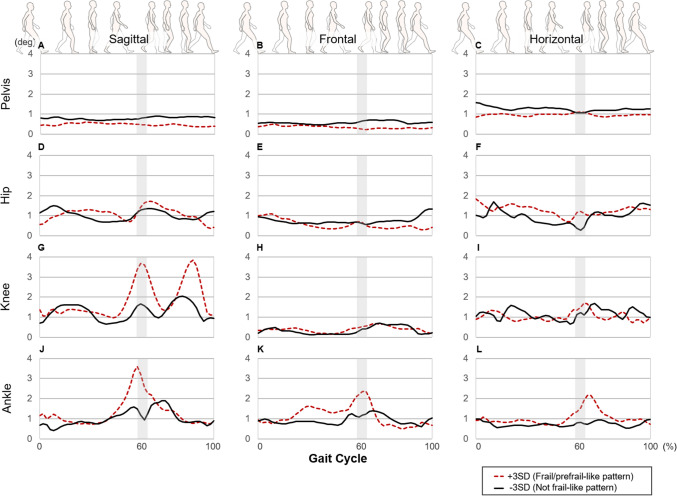
Waveforms of variability (standard deviation: SD) recombined from the principal component scores of principal component vectors 6 and 9. The gray highlighted area indicates the instance of the toe off (the transition from the stance phase to the swing phase). This area has a certain width because we did not separate the stance phase from the swing phase in the time-normalization procedure.

**Figure 2:**
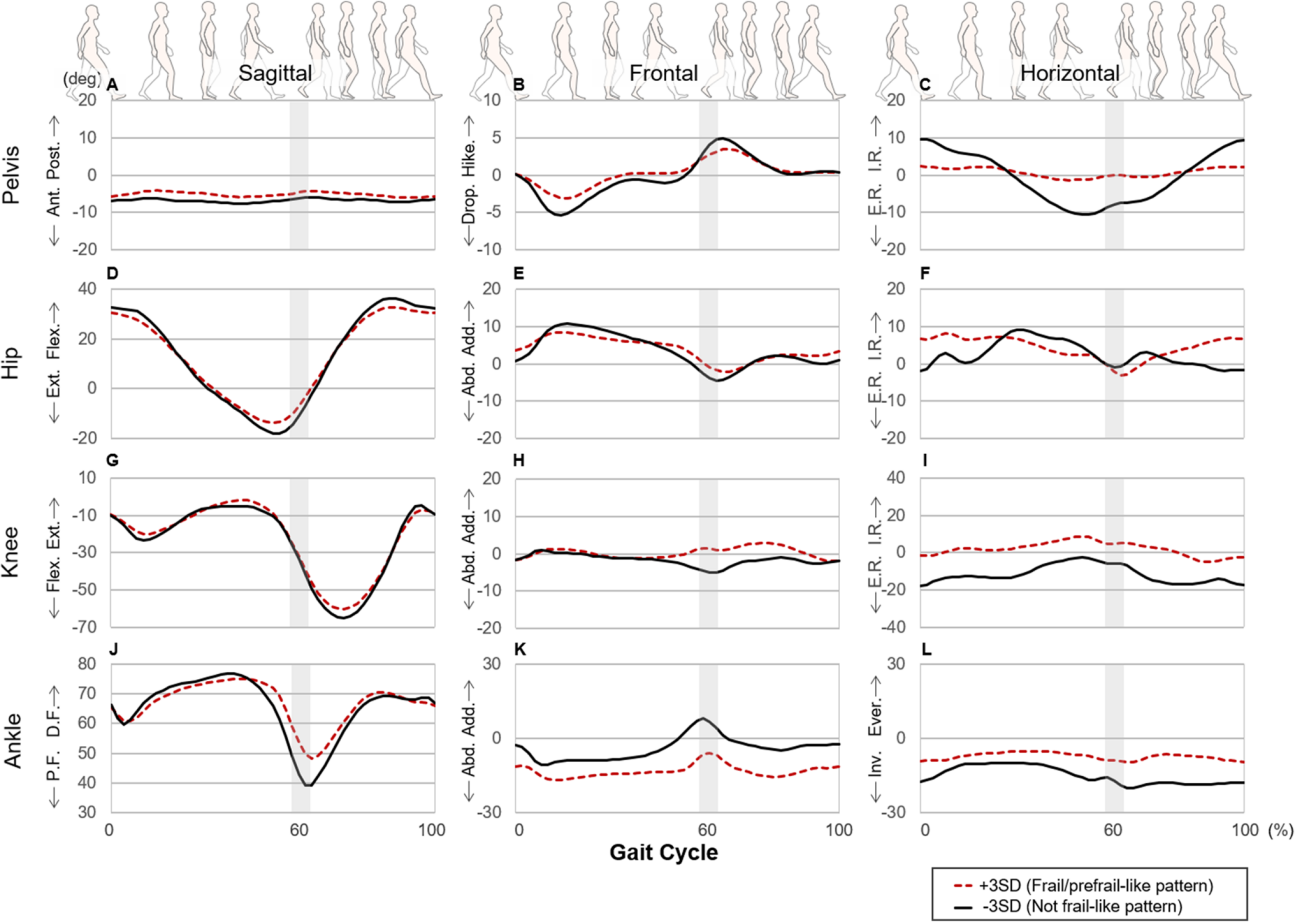
Waveforms of central tendency (average) recombined from the principal component scores of principal component vectors 6 and 9. The definitions of the abbreviations in the variability graph are as follows: *Post.* posterior tilt, *Ant.* anterior tilt, *Flex.* flexion, *Ext.* extension, *D.F.* dorsiflexion, *P.F.* plantarflexion, *Hike.* pelvic hike, *Drop.* pelvic drop, *Add.* adduction, *Abd.* abduction, *I.R*. internal rotation, *E.R.* external rotation, *Ever.* eversion, *Inv.* inversion.

We used these vectors to reconstruct joint kinematic waveforms (average and SD) of the pelvis, hip-, knee-, and ankle-joint angles on the sagittal, frontal, and horizontal planes (Figs. 1, 2). The reconstructed waveforms illustrated the differences between frail/prefrail participants (solid line) and non-frail participants (dotted line). As shown in Fig. 1, compared with non-frail participants, frail/prefrail participants tended to exhibit greater variability of the knee joint angle during the pre-swing, initial swing and terminal swing phases, and tended to exhibit greater variability of ankle joint angle during the pre-swing and initial swing phases in the sagittal plane. We used univariate analysis to confirm the presence of a significant difference between groups in the gait phase and joints where these trends were observed. Compared with the variability in the non-frail participants’ raw data, the raw data of frail/prefrail participants showed significantly greater variability of the knee, and ankle joints during the pre-swing and initial swing phases in the sagittal plane, and significantly greater variability of the knee joint during the terminal swing phase in the sagittal plane (see Supplementary Fig. S1). Furthermore, the reconstructed waveforms (Fig. 2) showed that frail/prefrail participants tended to exhibit smaller pelvis front and horizontal plane movement, hip extension, knee flexion and ankle plantar-flexion angles during all phases. Compared with the raw range of motion data (max angle minus minimum angle) for non-frail participants during the entire gait cycle, the raw data for frail/prefrail participants revealed a significantly smaller range of motion of the pelvis in the horizontal plane and of the hip joint in the sagittal plane (see Supplementary Fig. S2).

Table 3 shows the central tendency and the variability (coefficient of variation) of the spatiotemporal parameters. Univariate analysis revealed that frail/prefrail participants walked significantly more slowly (*p* = 0.045, d = 0.75) with a significantly shorter stride length (*p* = 0.003, d = 0.53) compared with non-frail participants. In addition, walking in frail/prefrail participants exhibited greater variability of stride time (*p* = 0.029, d = 0.58) and stance time percentage (*p* = 0.008, *d* = 0.72) compared with that in non-frail participants.

**Table 3:** Results of the central tendency and the variability (coefficient of variation) of the spatiotemporal parameters.

Variables		Non-frail (n = 30)	Frail/Prefrail (n = 30)	*p*-value	Cohen’s *d*
		(Mean ± SD)	(Mean ± SD)		
Walking speed (m/s)	Central tendency	1.43 ± 0.14	1.35 ± 0.16	0.04*	0.53
	Variability	2.50 ± 1.14	2.28 ± 0.94	0.42	0.21
Stride length (m)	Central tendency	1.32 ± 0.09	1.23 ± 0.14	0.00*	0.75
	Variability	1.60 ± 0.73	1.84 ± 1.08	0.31	0.26
Step width (m)	Central tendency	0.09 ± 0.02	0.08 ± 0.02	0.48	0.18
	Variability	21.5 ± 12.5	19.2 ± 9.88	0.42	0.21
Stride time (s)	Central tendency	0.93 ± 0.06	0.91 ± 0.05	0.21	0.33
	Variability	1.46 ± 0.73	1.96 ± 0.97	0.03*	0.58
Stance time (s)	Central tendency	0.55 ± 0.04	0.54 ± 0.03	0.42	0.21
	Variability	1.95 ± 0.83	2.56 ± 1.49	0.06	0.50
Swing time (s)	Central tendency	0.39 ± 0.02	0.38 ± 0.03	0.11	0.41
	Variability	2.16 ± 0.93	3.06 ± 2.26	0.05	0.52
Stance time percent (%)	Central tendency	58.6 ± 1.35	58.9 ± 1.23	0.27	0.29
	Variability	1.12 ± 0.59	1.72 ± 1.04	0.00*	0.72

The “*” symbol indicates significant differences between the non-frail and frail/prefrail groups (*p < 0.05).

The Pearson’s product moment correlation coefficients between the spatiotemporal parameters and principal component vectors are shown in Table 4. The correlation coefficients between the spatiotemporal parameters and principal component vectors 6 and 9 (the principal component vectors related to frailty) revealed that the principal component score of vector 6 was significantly correlated with the central tendencies of walking speed (*r* =  − 0.349, *p* = 0.006), step length (*r* =  − 0.428, *p* = 0.001) and stance time percentage (*r* = 0.282, *p* = 0.029), as well as variability of stride time (*r* = 0.269, *p* = 0.038). Vector 9 was significantly correlated with the variability of stance time percentage (*r* = 0.259, *p* = 0.046) (Table 4).

**Table 4:** Correlation coefficients between the spatiotemporal parameters and principal component vectors.

PCV	Walking speed (central tendency)		Stride length (central tendency)		Stride time (central tendency)		Stance time percent (central tendency)	
	*r*	*p*-value	*r*	*p*-valu	*r*	*p*-valu	*r*	*p*-valu
PCV1	− 0.10	0.44	− 0.08	0.55	0.09	0.48	0.29	0.02*
PCV2	0.28	0.03*	0.40	0.00*	0.08	0.52	− 0.14	0.29
PCV3	− 0.34	0.01*	− 0.22	0.09	0.23	0.08	0.26	0.05*
PCV4	0.21	0.11	0.27	0.04*	0.01	0.97	− 0.08	0.55
PCV5	0.20	0.12	0.19	0.14	− 0.11	0.40	− 0.03	0.83
PCV6	− 0.35	0.00*	− 0.43	0.00*	− 0.06	0.65	0.28	0.03*
PCV7	0.11	0.41	0.03	0.81	− 0.10	0.44	0.01	0.93
PCV8	− 0.14	0.29	− 0.19	0.16	− 0.05	0.70	− 0.24	0.07
PCV9	− 0.05	0.70	− 0.14	0.31	− 0.11	0.42	0.16	0.22
PCV10	0.07	0.60	0.03	0.83	− 0.05	0.70	− 0.01	0.95
PCV11	0.07	0.61	0.02	0.88	− 0.11	0.39	− 0.07	0.61
PCV12	− 0.19	0.15	− 0.11	0.40	0.22	0.10	0.43	0.00*
PCV13	− 0.22	0.10	− 0.22	0.09	0.05	0.69	0.14	0.28
PCV14	0.09	0.48	− 0.06	0.65	− 0.24	0.07	− 0.09	0.49
PCV15	− 0.29	0.03*	− 0.29	0.02*	0.09	0.49	0.02	0.90
PCV16	− 0.24	0.06	− 0.29	0.03*	− 0.06	0.67	0.36	0.01*
PCV17	0.27	0.04*	0.11	0.40	− 0.37	0.00*	0.09	0.52
PCV18	0.01	0.96	0.03	0.82	0.09	0.49	0.03	0.83
PCV19	0.00	0.99	0.07	0.58	0.16	0.21	− 0.09	0.49
PCV20	− 0.02	0.88	0.00	0.98	− 0.01	0.96	0.04	0.75
PCV21	− 0.11	0.39	− 0.12	0.36	− 0.06	0.67	0.01	0.96
PCV22	− 0.01	0.97	0.07	0.61	0.10	0.43	− 0.24	0.06
PCV23	− 0.23	0.08	− 0.09	0.52	0.34	0.01*	− 0.11	0.40
PCV24	0.10	0.47	− 0.05	0.74	− 0.14	0.30	0.02	0.88
PCV25	0.18	0.16	0.14	0.30	− 0.13	0.34	0.03	0.81
PCV26	− 0.07	0.60	0.09	0.50	0.19	0.15	0.01	0.97

PCV	Walking speed (variability)		Stride length (variability)		Stride time (variability)		Stance time percent (variability)	
	*r*	*p*-value	*r*	*p*-valu	*r*	*p*-valu	*r*	*p*-valu
PCV1	0.15	0.26	0.26	0.05*	0.05	0.71	0.13	0.31
PCV2	− 0.13	0.31	− 0.03	0.81	0.24	0.06	0.04	0.78
PCV3	0.37	0.00*	0.26	0.04*	0.43	0.00*	0.00	0.99
PCV4	− 0.23	0.08	− 0.21	0.11	− 0.15	0.25	− 0.03	0.81
PCV5	− 0.11	0.39	− 0.22	0.09	0.25	0.06	− 0.19	0.15
PCV6	0.15	0.26	0.22	0.09	0.27	0.04*	0.19	0.14
PCV7	− 0.12	0.38	− 0.20	0.12	− 0.12	0.37	− 0.19	0.15
PCV8	0.16	0.22	0.05	0.74	0.12	0.37	0.28	0.03*
PCV9	− 0.20	0.12	0.20	0.06	0.02	0.89	0.26	0.04*
PCV10	0.25	0.05	0.00	1.00	0.09	0.51	− 0.17	0.20
PCV11	− 0.13	0.32	− 0.13	0.33	− 0.02	0.91	− 0.12	0.37
PCV12	0.02	0.91	− 0.15	0.26	0.06	0.64	− 0.06	0.66
PCV13	0.02	0.91	0.08	0.52	0.15	0.24	0.24	0.06
PCV14	− 0.07	0.59	− 0.02	0.91	− 0.10	0.44	0.11	0.42
PCV15	− 0.09	0.52	0.16	0.22	0.28	0.03*	0.30	0.02*
PCV16	0.09	0.48	0.13	0.32	0.05	0.73	− 0.07	0.62
PCV17	0.05	0.72	− 0.04	0.79	0.13	0.34	0.15	0.25
PCV18	0.03	0.82	− 0.07	0.57	0.05	0.73	0.18	0.17
PCV19	0.20	0.13	− 0.09	0.51	0.06	0.66	0.03	0.82
PCV20	0.13	0.32	0.11	0.42	0.10	0.45	0.18	0.16
PCV21	− 0.12	0.35	0.34	0.01*	0.02	0.90	0.28	0.03*
PCV22	− 0.16	0.24	0.09	0.50	0.00	0.97	− 0.03	0.81
PCV23	− 0.27	0.04*	0.05	0.73	− 0.05	0.69	0.21	0.11
PCV24	0.05	0.69	− 0.04	0.78	− 0.04	0.79	− 0.05	0.68
PCV25	− 0.30	0.02*	− 0.15	0.26	− 0.17	0.19	0.07	0.61
PCV26	0.06	0.65	0.00	0.98	− 0.18	0.16	− 0.14	0.29

We calculated the Pearson’s product-moment correlation coefficients between the principal component vectors that were related to frailty status and the spatiotemporal parameters.

*PCV* principal component vector, *r* Pearson’s product-moment correlation coefficients.

The “*” symbol indicates significant correlations (*p < 0.05).

## Discussion

The purpose of the current study was to compare the key joint kinematics characteristics during the entire gait cycle in frail and non-frail older women using principal component analysis. Principal component analysis was conducted on the time-normalized average and SD of the pelvis and lower limb joint angles. Significant differences between frailty status groups were found for two principal component vectors: vector 6 and vector 9. Vector 6 had a moderate effect (*p* = 0.024, *d* = 0.60) and vector 9 had a large effect (*p* = 0.001, *d* = 0.72), which were then used to reconstruct kinematic waveforms of the joint angles. Because the principal component scores for the two vectors were both positive on average for the frail/prefrail group and negative on average for the non-frail group, the reconstructed waveforms with positive and negative deviations from the mean can be interpreted as the extreme gait characteristics of frail/prefrail and non-frail participants, respectively.

Importantly, the reconstructed waveforms in the current study indicated that the gait characteristics of frail/prefrail participants exhibited greater within-participant variability in the knee- and ankle-joint angles compared with non-frail participants (see Fig. 1). This greater variability in the knee- and ankle-joint angles of frail/prefrail participants compared with that of non-frail participants could potentially contribute to increased variability in spatiotemporal parameters. In accord with this possibility, a significant positive correlation was found between the principal component scores of the principal component vectors used for reconstruction of waveforms and the variability of stride time (r = 0.269) as well as the variability of stance time percentage (r = 0.259) (see Table 4). Regarding spatiotemporal parameters, the variability of stride time and stance time percentage were significantly greater in frail/prefrail participants (an increase of approximately 1.3 times compared with those in non-frail participants; Table 3). Previous studies also reported greater variability in spatiotemporal parameters in frail older adults^17,18,24,25^. Two studies using the same definition of frailty status as that used in the current study examined a combined sample of both males and females^17,18^. First, Montero-Odasso et al.^17^ reported that variability of stride time was approximately 1.3 times greater in prefrail older adults compared with that in non-frail older adults, and approximately 1.6 times greater in frail older adults. Second, Ritt et al.^18^ reported that variability of stride time was approximately 1.3 times greater in prefrail and frail older adults compared with that in non-frail older adults. These findings are similar to the increased variability of stride time in frail/prefrail participants found in the current study. These results suggest that, in frail older women, the variability in spatiotemporal parameters (stride time, stance time percentage) may increase because of greater variability in the knee- and ankle-joint angles during walking. This increased variation in joint angles was not observed in the pelvis or hip joints (see Fig. 1). Although they did not examine frail older people specifically, previous studies have reported that aging is associated with decreased muscle activity during walking and decreased proprioception in distal areas compared with that in proximal areas^43,44^. One previous study of frail older adults reported that gait variability of frail older adults was associated with quadriceps quality^45^. Therefore, we speculate that these aging and frailty-related phenomena may affect the knee and ankle joints, possibly increasing the variability of joint angle in frail participants.

The reconstructed average joint angle waveforms revealed that frail/prefrail participants tended to exhibit smaller pelvis front and horizontal plane movement, hip extension, knee flexion and ankle plantar-flexion angles (see Fig. 2). These observations are well-known characteristics among older people with slow walking speed^46–48^. In the current study, the walking speed of frail/prefrail participants was slower than that of non-frail participants (non-frail: 1.43 m/s, frail/prefrail: 1.35 m/s, Table 3). These overall walking speeds were faster than those reported in a previous study that used the same definition of frailty and included female participants only, but shows the same trend of slower speed in frail/prefrail participants compared with that in non-frail participants (non-frail: 0.95 m/s, frail/prefrail: 0.62 m/s)^49^. In addition, several previous studies that included both males and women reported a similar trend (non-frail: 1.03–1.4 m/s, prefrail; 0.92–1.4 m/s, frail: 0.64–1.30 m/s,^17,19,28,50–54^. Significant correlations with walking speed were found in the principal component scores of vectors 2, 3, 6, 15 and 17 (Table 4). Among these vectors, only vector 6 exhibited a significant difference between frailty status groups. Therefore, we concluded that the joint kinematics features related to vector 6 were the joint kinematics features associated with frailty. Thus, we reconstructed a waveform with principal component vector 6 and produced waveforms for frail/prefrail participants with smaller pelvis front and horizontal plane movement, hip extension, knee flexion and ankle plantar-flexion angles (see Supplementary Fig. S4). On the basis of these results, we hypothesize that these decreases in joint angle during walking could lead to a decrease in the stride length regardless of frailty status, leading to slower walking speed. Slow walking speed has also been adopted as one of the criteria for the definition of frailty by Fried et al.^6^, and has been reported to provide a good reflection of frailty status^6,19–22^. It is therefore not surprising that frail older women exhibited a slower walking speed than non-frail older women in the current study. Moreover, because walking speed is related to joint angle, the finding that frail older women exhibited a significantly lower joint angle than non-frail older women may be expected. Among the principal component vectors associated with frailty, walking speed was significantly correlated with principal component vector 6, but not with vector 9 (see Table 4). This result indicates that only the joint kinematics related to principal component vector 6, and not those related to vector 9, affected gait speed. Reconstructing a waveform with only vector 9 reduced the differences in joint angles between frail/prefrail participants and non-frail participants that were observed in Fig. 2, which was reconstructed with vectors 6 and 9, and Supplementary Fig. 4, which was constructed with vector 6 only (see Supplementary Fig. S6). In addition, the waveform reconstructed with only vector 9 exhibited greater within-participant variability in the knee- and ankle-joint angles of frail/prefrail participants compared with the other waveforms (see Supplementary Fig. S5). Therefore, knee and ankle joint variability (the joint kinematics features related to vector 9) may capture the gait characteristics of frail older women that are not affected by the change in walking speed associated with the frailty status of participants.

These types of variability can be measured using several easy-to-use ambulatory devices, in addition to 3D motion analysis systems. One previous study proposed a new ambulatory measurement method for measuring 3D knee joint angle during walking using two wearable inertial measurement units, each of which consists of a tri-axial gyroscope and an accelerometer^55^. Other studies reported that measurement using wearable inertial motion sensors and in-shoe pressure sensors provided a reliable method for measuring lower extremity joint kinetics during walking^56^. The variability of gait reflects complex physiological changes, such as neural control, muscle function, postural control and cardiovascular function^57^. Thus, the large joint angle variability observed in the current study may reflect physiological changes in frail older women. The change from a healthy state to a frail state in older people could potentially be detected early by observing joint variability. The measurement of frailty-related joint angle variability could be performed easily at health checkups, community clinics, or at home. Simple measurement methods introduced in previous studies could be applied to older adults, which would be useful for elucidating the physiological changes associated with frailty and establishing systems for early detection. Further research will be needed to develop such methods in the future.

### Limitations

The present study involved several limitations that should be noted. First, soft tissue artifacts may have caused a bias in the observed plane angles, particularly in the hip and knee joints. Although we placed markers on the body’s bony landmarks, the existence of such artifacts should be considered. Second, the participants in the current study were 30 asymptomatic older women with relatively fast gait speeds (1.39 m/s on average). Thus, the findings may not be generalizable to some patient populations or target groups. Nevertheless, the current findings provide an important basis for further validation of methods for identifying the characteristic features of joint kinematics during walking that are associated with frailty in different target groups. Further data accumulation and analyses from various perspectives, such as the dominant and non-dominant legs and asymmetry, may provide new insights to inform the development of an accurate model for distinguishing frailty and the trajectory of frailty-related gait deterioration among older adults, particularly those with diseases or disorders.

## Conclusions

In the current study, principal component analysis was used to identify the key characteristic features of joint kinematics of gait related to frailty status. Statistical analyses revealed that only principal component vectors 6 and 9 were related to frailty. We recombined the joint kinematics corresponding to these vectors and found greater variability of knee- and ankle-joint angles in the sagittal plane while walking in frail older women, compared with that in non-frail older women. Therefore, these results suggest that greater variability of the knee- and ankle-joint angles in the sagittal plane are characteristic features of joint kinematics during walking in frail older women. Depending on the findings of future validation studies, the measurement of variability may constitute part of a useful tool for evaluating frailty in both clinical and research settings.

## Methods

### Participants

Thirty healthy community-dwelling older females aged 60 and older who were able to walk independently were recruited for the study. Participants were excluded if they: (1) needed assistive devices (e.g., canes, crutches, or orthotic devices); (2) underwent surgery for trauma or orthopedic diseases; (3) had neurological disorders; and/or (4) regularly engaged in competitive sports on a professional level. All of the participants were capable of walking independently without assistive devices, had normal or corrected-to-normal vision, and had no diseases that they were aware of. Participants were asked to maintain their normal dietary habits and refrain from vigorous physical activity the day before and immediately before the experiment. The experimental protocol was approved by the ethical review board at the National Institute of Advanced Industrial Science and Technology (AIST), and all participants gave written informed consent before participating. The research complied with the principles of the Declaration of Helsinki.

### Definition of frailty

Many definitions of frailty have been proposed. Fried et al. used five criteria (weight loss, slowness, weakness, exhaustion, and low activity) to identify frailty^6^. Fried’s phenotype criteria are the most widely cited criteria and focus on physical frailty^25,58–60^. Rockwood et al. developed a frailty index based on impairments in cognitive status, mood, motivation, communication, mobility, balance, bowel and bladder function, activities of daily living, nutrition, social resources and number of comorbidities^61^. Mitnitski et al. constructed a frailty index based on 20 deficits as observed in a structural clinical examination based on the comprehensive geriatric assessment (CGA)^62^. Jones et al. also based their frailty index based on the CGA, which included 10 standard domains to construct a three-level frailty index permitting risk stratification of future adverse outcomes^63^. In addition, Chin et al. compared three different working definitions of frailty: inactivity plus low energy intake, inactivity plus weight-loss and inactivity plus low body mass index^64^. Although many definitions of frailty have been proposed, because the current study was focused was on kinematic characteristics during walking and frailty status, we used Fried’s frailty criteria (the revised Japanese version)^6,65^, which is the most widely used frailty phenotype assessment tool. We assessed weight loss by asking a single “yes or no” question, “Have you lost 2–3 kg or more of your body weight in the past 6 months?” Slowness was established using a cutoff of < 1.0 m/s. We assessed weakness using maximum grip strength using a Smedley-type dynamometer (TKK 5401; Takei Scientific Instruments Co., Tokyo, Japan) and a sex-specific cutoff (< 26 kg for males and < 18 kg for females). We assessed exhaustion with a “yes or no” question: “In the last 2 weeks, have you felt tired without a reason?” We assessed low activity using the following two questions: “Do you engage in moderate levels of physical exercise or sports aimed at health?” and “Do you engage in low levels of physical exercise aimed at health?” If participants responded “No” to these two questions, they were classified as having low physical activity. Participants whose responses did not correspond to any of these target criteria were considered to be robust, those who met one or two criteria were considered prefrail, and those who met three or more criteria were considered frail. In this model, we combined frail and prefrail participants into a frail/prefrail group because of the limited number of frail participants.

### Definition of sarcopenia

Although several criteria have been proposed to define sarcopenia, the recently reported criteria of the Asian Working Group for Sarcopenia (AWGS 2019) were applied in this study^66^. The skeletal muscle mass index was measured using dual energy X-ray absorptiometry with a bioelectrical impedance data acquisition system (Inbody 770; Biospace Co, Ltd, Seoul, Korea). Low muscle mass was defined as a skeletal muscle index value of less than 7.0 kg/m^2^ in males and less than 5.7 kg/m^2^ in females. Low muscle strength was defined as handgrip strength < 28 kg for males and < 18 kg for females. Criteria for low physical performance was 6-m walk < 1.0 m/s. Sarcopenia was defined as “low muscle mass + low muscle strength or low physical performance”, and severe sarcopenia was defined as “low muscle mass + low muscle strength and low physical performance”.

### Gait measurement

Gait measurement was performed in a room with a straight 10-m path on which the participants could walk. All participants wore the same type of clothing during the experiment (sleeveless shirt and spats), which was provided by the experimenter. The clothing size was selected by participants. During gait measurement, participants were asked to walk barefoot at a comfortable, self-selected speed. Three-dimensional (3D) positional data were obtained during walking, using reflective markers and 10-camera and a 3D motion capture system (MAC3D, Motion Analysis Corporation, California, USA) with a 200 Hz sampling frequency. A total of 57 infrared reflective markers were attached in accordance with the guidelines of the Visual 3D software (C-Motion Inc., Rockville, MD, US). Simultaneously, ground reaction forces (GRFs) were obtained using four force plates (BP400600-2000, AMTI, Watertown, MA, US) sampled at 2000 Hz. Before the walking trials, the positions of the markers were recorded while participants stood stationary. The participants were then given sufficient practice walking to ensure a natural gait. After the practice, five successful trials per each leg were recorded, in which each participant correctly stepped on a force plate.

### Data analysis

The raw motion and GRF data were digitally filtered using a zero-lag, fourth-order, low-pass Butterworth filter; the filter cut-off frequencies were 10 Hz for the positional data and 56 Hz for the GRF data based on a previous study^67^. The angles of the hip, knee, and ankle joints, and the pelvis-link angle during one gait cycle were calculated for the x-axis (i.e., flexion–extension), y-axis (i.e., abduction–adduction), and z-axis (i.e., internal–external rotation) using a Cardan sequence of rotations (X–Y-Z) from the trajectories measured in each trial. Based on a previous study^40^, the angles were time-normalized using the gait cycle duration determined from the force plate data and divided into 51 variables ranging from 0 to 100%. Therefore, each trial corresponded to a dataset of 612 variables (51 time points, four angles in three axes). Means and within-participants coefficients of variation of walking speed, stride length, step width, stride time, stance time, swing time, and stance time percentage were also determined, to help understand the gait characteristics. Low-pass filtering, variable calculation (i.e., joint and link angles, and spatiotemporal parameters), and time normalization processes were performed using Visual 3D software.

### Statistical analysis

Statistical analyses were performed using the Statistical Package for the Social Sciences version 15.0 (SPSS Inc., USA). Principal component analysis is a multivariate statistical technique for analyzing data waveforms based on their variance, and has been used in various biomechanical studies to identify the movement characteristics of various groups under various conditions^33–37^. We therefore expected that principal component analysis analyses could be used to determine the relationship between the gait patterns observed during normal walking, as represented by the principal component vectors, and the states of frailty. The specific principal component analysis procedure and kinematic waveform reconstruction were conducted using the method reported by Kobayashi et al.^38–42^. Briefly, in our study, the following six steps were used to conduct the principal component analysis. First, the the intra-participant average and SDs were calculated for each time point within the five trials of data obtained from each leg of each participant. Second, mean centering was conducted on each of 1224 variables (i.e., averages and SDs for 51 time points, four angles in three axes) using the z-score:

$$ {\text{zt }} = \, \left( {{\text{Xt}} - \upmu {\text{t}}} \right)/\upsigma {\text{t}}, $$

where zt is the z-score for the parameter t, Xt is the raw data of the parameter t, μt is the mean of the parameter t for the participant, and σt is SD of the parameter t. Third, input matrices of 60 data points (each leg of 30 participants) by 1224 variables were constructed. Fourth, principal component vectors were extracted until their cumulative ratio attained 84% of the total variance. Fifth, the statistical analyses described in the next subsection were conducted to identify the main effects of frailty status on the joint kinematic characteristics represented by the principal component vectors. Finally, for each principal component vector, simulated kinematic waveforms were reconstructed from the principal component scores with very large or very small values (deviating from the mean by three SDs), to interpret data on the average joint angle and joint angle variability corresponding to the principal component vectors. If there were significant differences between the principal component scores obtained from frail/prefrail participants and those obtained from non-frail participants for a certain principal component vector, the joint kinematics corresponding to this vector were interpreted as key characteristic features of joint kinematics of walking that are related to the states of frailty. Univariate analysis was conducted on the principal component scores between two groups using independent t-tests, in the same way that the differences between the principal component vectors of different groups were tested in previous studies^34,35^. Furthermore, we also calculated Cohen’s d effect size to validate the results of the t-test according to the recommendation by Lakens^68^ and interpreted the results using 0.2 for a small effect, 0.5 for a moderate effect, and 0.8 for a large effect, according to previously reported guidelines^69^. In addition, we conducted independent t-tests to analyze differences (central tendencies and variability) between the following parameters: walking speed, stride length, step width, stride time, stance time, swing time, and stance time percentage. Furthermore, we determined the Pearson’s product-moment correlation coefficients between the principal component vector related to the states of frailty and the above-mentioned parameters. Differences were considered statistically significant when *p*-values were < 0.05.

## Supplementary Information

Supplementary Figures.

## Data Availability

All data needed to evaluate the conclusions in the paper are present in the paper, Supplementary Material, and/or references cited within.
