# Supplementary material for: Kinematic characteristics during gait in frail older women identified by principal component analysis
Source: Sci Rep. 2022 Jan 31;12:1676. doi: 10.1038/s41598-022-04801-2 (PMC8803892; doi:10.1038/s41598-022-04801-2)
Supplement: Supplementary file 1 — Supplementary Figures. [file 41598_2022_4801_MOESM1_ESM.pdf]

Kinematic characteristics during gait in frail older women identified by principal component analysis

Authors:

Wakako Tsuchida,[1*,] Yoshiyuki Kobayashi,[2*,] Koh Inoue,[3,] Masanori Horie,[1,] Kumiko Yoshihara,[1,] Toshihiko Ooie[1*,]

Affiliations:

[1]Health and Medical Research Institute, Department of Life Science and Biotechnology, National Institute of Advanced Industrial Science and Technology (AIST), 2217-14 Hayashi-cho, Takamatsu, Kagawa, 761-0395, Japan

[2] Human Augmentation Research Center, National Institute of Advanced Industrial Science and Technology (AIST), Kashiwa II Campus, University of Tokyo, 6-2-3 Kashiwanoha, Kashiwa, Chiba 277-0882 Japan

[3] Kagawa University Faculty of Engineering and Design, Hayashi-cho 2217-20, Takamatsu, Kagawa, 761-0396, Japan.

*Correspondence to: w-tsuchida@aist.go.jp, kobayashi-yoshiyuki@aist.go.jp, toshihiko-ooie@aist.go.jp

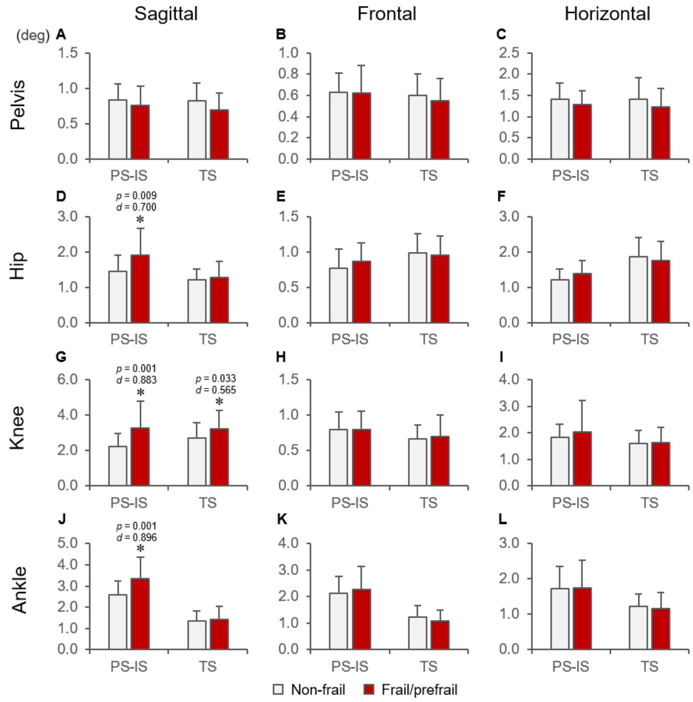

Supplementary figure 1. The peak variability (standard deviation [SD]) of joint angle between the pre-swing and the initial swing phase (PS-IS), and in the terminal swing phase (TS). Data are expressed as means ± SD. The data were analyzed using univariate analysis (independent t-tests) for comparisons between the non-frail and frail/prefrail groups. The "*" symbol indicates significant differences between the two groups (* $p < 0.05$). P-values and the effect sizes (Cohen's d) are shown.

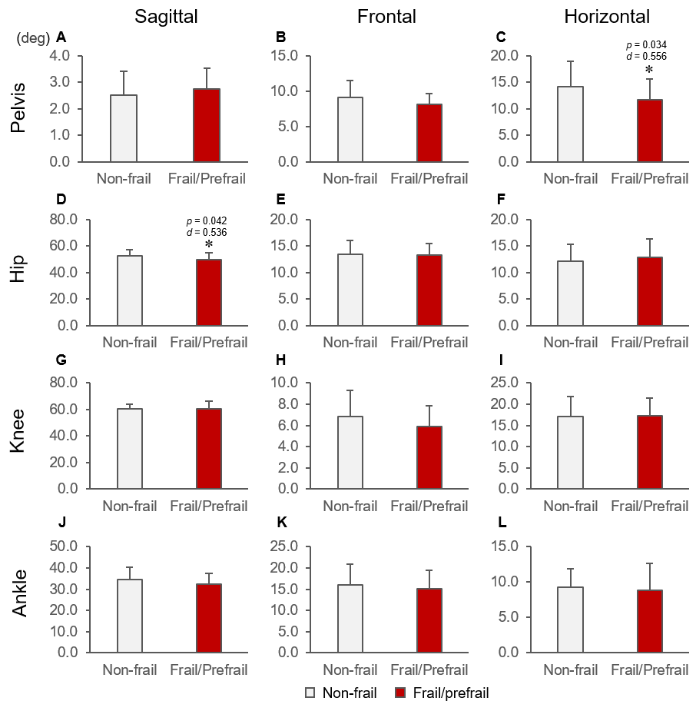

Supplementary figure 2. The range of motion (joint angle) during one walking cycle. The ranges of motion were calculated as the maximum angle minus the minimum angle during one gait cycle. Data are expressed as means ± SD. The data were analyzed using univariate analysis (independent t-tests) for comparisons between the non-frail and frail/prefrail groups. The "*" symbol indicates significant differences between the two groups (* $p < 0.05$). P-values and effect sizes (Cohen's d) are shown.

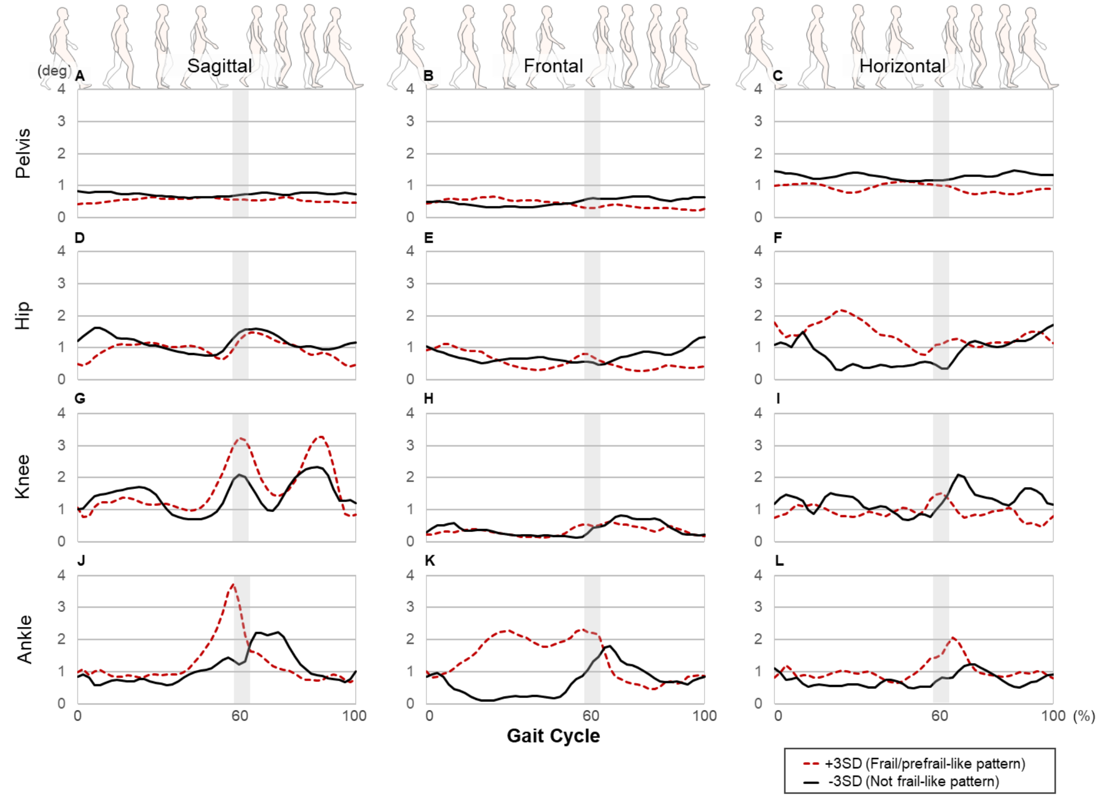

Supplementary figure 3. Waveforms of variability (standard deviation: SD) recombined from the principal component scores of principal component vector 6. The gray highlighted area indicates the instance of the toe off (the transition from the stance phase to the swing phase). This area has a certain width because we did not separate the stance phase from the swing phase in the time-normalization procedure.

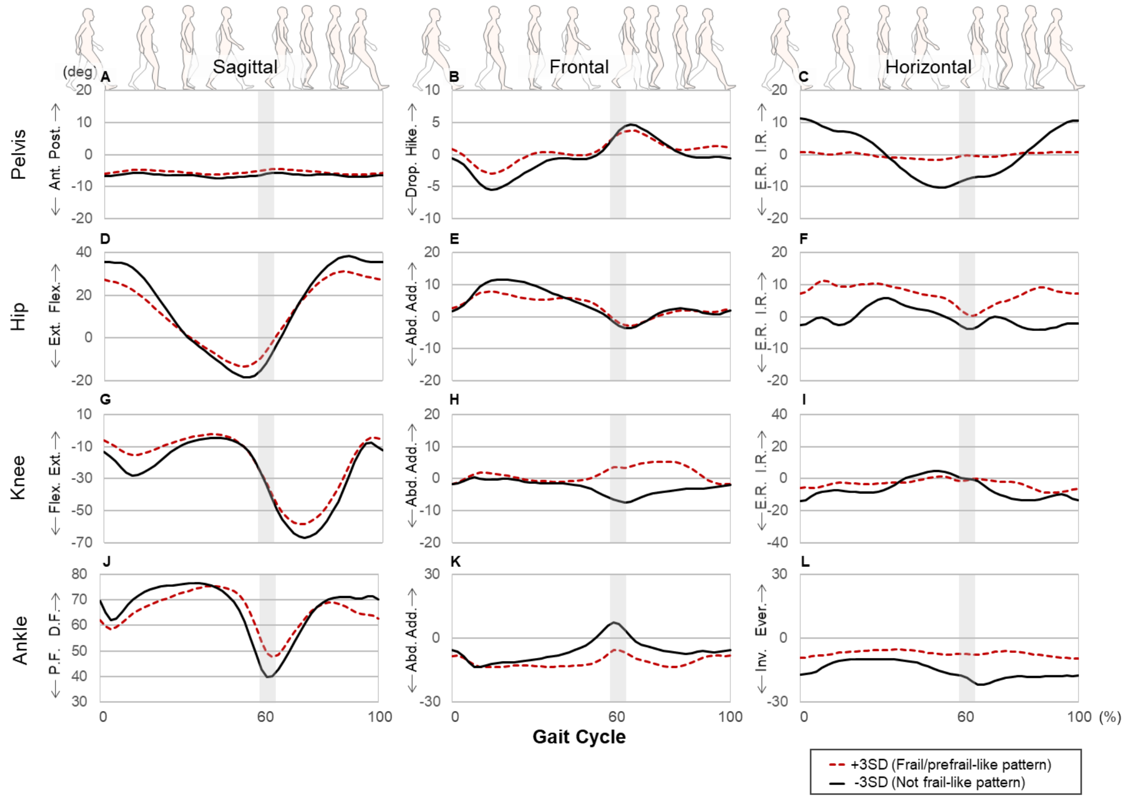

Supplementary figure 4. Waveforms of central tendency (average) recombined from the principal component scores of principal component vector 6. The definitions of the abbreviations in the graph are as follows: Post.: Posterior tilt, Ant.: Anterior tilt, Flex.: Flexion, Ext.: Extension, D.F.: Dorsiflexion, P.F.: Plantarflexion, Hike.: Pelvic hike, Drop.: Pelvic drop, Add.: Adduction, Abd.: Abduction, I.R.: Internal rotation, E.R.: External rotation, Ever.: Eversion, Inv.: Inversion.

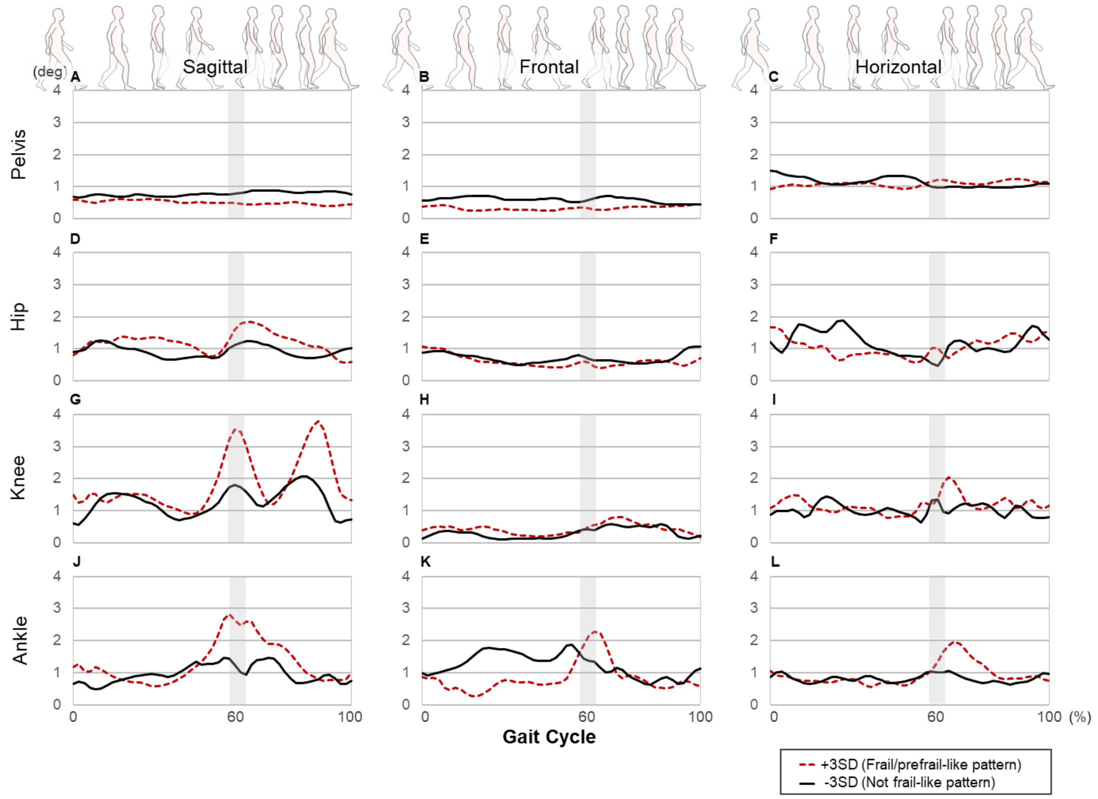

Supplementary figure 5. Waveforms of variability (standard deviation: SD) recombined from the principal component scores of principal component vector 9.

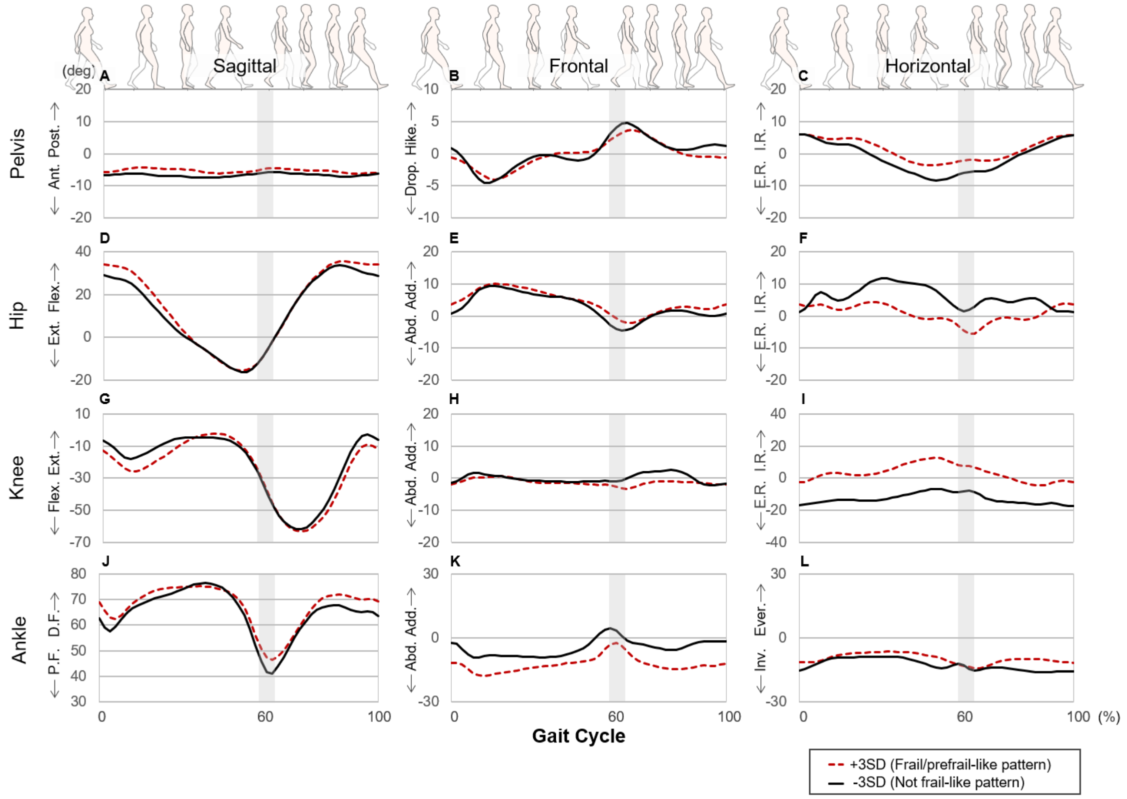

Supplementary figure 6. Waveforms of central tendency (average) recombined from the principal component scores of principal component vector 9. The definitions of the abbreviations in the variability graph are as follows: Post.: Posterior tilt, Ant.: Anterior tilt, Flex.: Flexion, Ext.: Extension, D.F.: Dorsiflexion, P.F.: Plantarflexion, Hike.: Pelvic hike, Drop.: Pelvic drop, Add.: Adduction, Abd.: Abduction, I.R.: Internal rotation, E.R.: External rotation, Ever.: Eversion, Inv.: Inversion.
